# Automatic aortic root landmark detection in CTA images for preprocedural planning of transcatheter aortic valve implantation

**DOI:** 10.1007/s10554-015-0793-9

**Published:** 2015-10-23

**Authors:** Mustafa Elattar, Esther Wiegerinck, Floortje van Kesteren, Lucile Dubois, Nils Planken, Ed Vanbavel, Jan Baan, Henk Marquering

**Affiliations:** Biomedical Engineering and Physics, Academic Medical Center, University of Amsterdam, Amsterdam, The Netherlands; Heartcenter, Academic Medical Center, University of Amsterdam, Amsterdam, The Netherlands; Radiology, Academic Medical Center, University of Amsterdam, Amsterdam, The Netherlands; Biomedical Engineering, Polytech Lyon, Université Claude Bernard Lyon, Villeurbanne, France

**Keywords:** CTA, TAVI, Landmarks, Detection, Aortic root, Segmentation

## Abstract

Transcatheter aortic valve implantation is currently a well-established minimal invasive treatment option for patients with severe aortic valve stenosis. CT Angiography is used for the pre-operative planning and sizing of the prosthesis. To reduce the inconsistency in sizing due to interobserver variability, we introduce and evaluate an automatic aortic root landmarks detection method to determine the sizing parameters. The proposed algorithm detects the sinotubular junction, two coronary ostia, and three valvular hinge points on a segmented aortic root surface. Using these aortic root landmarks, the automated method determines annulus radius, annulus orientation, and distance from annulus plane to right and left coronary ostia. Validation is performed by the comparison with manual measurements of two observers for 40 CTA image datasets. Detection of landmarks showed high accuracy where the mean distance between the automatically detected and reference landmarks was 2.81 ± 2.08 mm, comparable to the interobserver variation of 2.67 ± 2.52 mm. The mean annulus to coronary ostium distance was 16.9 ± 3.3 and 17.1 ± 3.3 mm for the automated and the reference manual measurements, respectively, with a mean paired difference of 1.89 ± 1.71 mm and interobserver mean paired difference of 1.38 ± 1.52 mm. Automated detection of aortic root landmarks enables automated sizing with good agreement with manual measurements, which suggests applicability of the presented method in current clinical practice.

## Introduction

Aortic stenosis is the most common valvular heart disease in the elderly population, with a prevalence of 2–7 % in patients older than 65 years [1–3]. Aortic stenosis is most frequently of calcific degenerative etiology, with extensive calcium accumulation on the aortic valve leaflets [4]. Advancing from the base of the cusps of the aortic valve to the leaflets, this slowly progressive disease eventually reduces leaflet motion and valve area [5].

Traditional treatment of severe aortic valve stenosis is aortic valve replacement (AVR) by open-heart surgery. Aortic valve replacements are the most common heart valve operations, accounting for 60–70 % of all valve surgeries performed in the elderly [6]. With a quarter of a million procedures performed annually, it is the most common valvular heart procedure [7]. However, at least 30 % of patients are not referred for AVR due to estimated high risk based on advanced age or presence of various comorbidities [8]. For these high risk patients, transcatheter aortic valve implantation (TAVI) is a less invasive procedure for the treatment of severe aortic valve stenosis. In TAVI, the prosthetic valve is inserted and deployed using a catheter through a small puncture of the femoral artery (the transfemoral approach), a small incision at the apex of the heart (the transapical approach) or directly through the aortic arch (transaortic approach) [9]. TAVI is however still associated with a number of adverse effects, such as paravalvular leakage, stroke, coronary obstruction, and conduction disorders [10]. CT Angiography (CTA) imaging plays an important role in pre-operative surgical planning and patient selection and can be used for post-operative outcome assessment [11]. Preprocedural assessment of patient eligibility and sizing parameters of the aortic root are both crucial to choose the suitable type of prosthesis as well as the prosthesis dimensions [12].

**Fig. 1:**
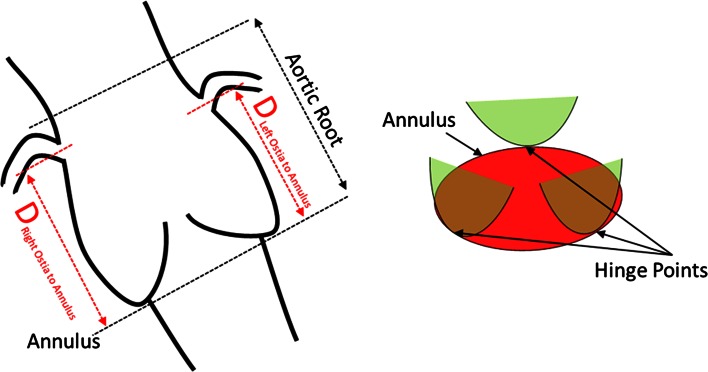
Schematic drawing for the different required measurements (*Left*) and the location of the hinge points in relation with leaflets (*Right*)

During the pre-procedure planning, several important sizing parameters of the aortic valve are indispensable. A number of commercial tools to assess these measurements have been introduced in the market, For example, there is validated software for the automated analysis of annulus minimal and maximal diameter, perimeter and area [13]. However, in the commercially available tools there is no standardized automated solution for the more complex measures such as annular plane to coronary ostium distance. This distance is a critical parameter for patient selection since a short distance increases the risk of blocking coronary ostia after valve deployment [14, 15]. (See Fig. 1). This study advances existing automated measurement by introducing a landmark-based detection method for more complex aortic root measurement. We hypothesized that automated aortic root landmarks detection would allow speeding up the measurements, standardize the planning, and reduce interobserver variation.

In this work, we introduce a fully automated algorithm for extraction of aortic root landmarks and calculation of sizing parameters in CTA images of patients eligible for TAVI. The accuracy of our approach is assessed and compared with the interobserver variation.

## Methods

**Fig. 2:**
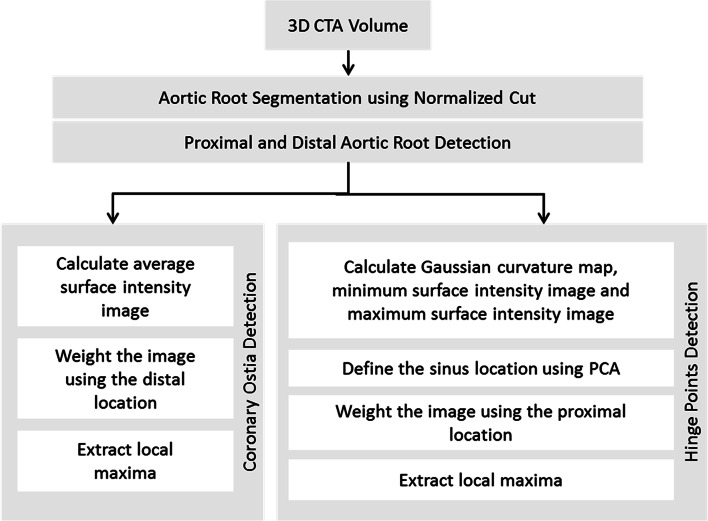
Schematic overview of the proposed algorithm

We propose an image analysis pipeline based on a segmented aortic root surface as illustrated in Fig. 2 [16]. This segmented aortic root surface is used as a 2D search space for finding the required landmarks. These landmarks are used for calculating sizing parameters required for the TAVI procedure. Each landmark is extracted based on specific characteristics after the estimation of the proximal and distal extents of the aortic root.

In the next sections, we describe the used image data, the aortic root surface segmentation, the landmarks detection methods, and the validation of the detected landmarks by comparison with the manual delineated landmarks.

### Image data

A dataset of thirty preprocedural 3D CTA volumes of TAVI patients with aortic stenosis and ten CTA volumes of non-stenotic patients from our institute (Academic Medical Center, The Netherlands) was used for validating our proposed algorithm. The dataset included seventeen females and twenty-three males. The average age of the stenotic patients was 82 years ranging from 68 to 93 years and the average age of the non-stenotic patients was 45 years ranging from 34 to 58 years.

The dynamics during the cardiac cycle may result in differences between systolic and diastolic measurements potentially influencing aortic root sizing in the preprocedural TAVI planning [17–21]. Therefore, we included ten end-systole volumes in addition to thirty end-diastole volumes to evaluate our proposed algorithm. For the latter, the acquisition at 70 % of the cardiac cycle was selected. This phase represents the end diastole phase in which the aortic valve is closed [22]. Ten patients were analyzed at the end systole phase at 30 % of the cardiac cycle.

The closed valve separates the aortic root lumen from the left ventricle outflow tract lumen, which is important for accurate aortic root detection [16]. All CT-scans were performed on a Philips Brilliance 64 slice CT scanner; imaging parameters were 120 kV, matrix 512, and convolution kernel B. The chest, abdomen, and pelvis were scanned using one bolus of 120 ml contrast Iomeron 400, intravenously infused at a rate of 5 ml/s. Image volumes contain 500–600 slices. The size of each slice in a volume is 512 × 512 pixels with a 16 bit depth. The in-plane image resolution is isotropic and varies from 0.44 to 0.68 mm. The slice thickness for all data sets is 0.9 mm with an overlap of successive slices of 0.45 mm.

### Sizing parameters

The sizing parameters assess the distances between six landmarks located on the aortic root surface; the sinotubular junction, the right coronary ostium, left coronary ostium, the right coronary hinge point, left coronary hinge point, and the non-coronary hinge point (see Fig. 1). The annulus to ostium distance is evaluated by calculating the annulus plane, which fits the three hinge points, and finding shortest distance from the plane to right and left ostium. The radius of the circle which fits the three hinge points is calculated.

### Aortic root surface segmentation

**Fig. 3:**
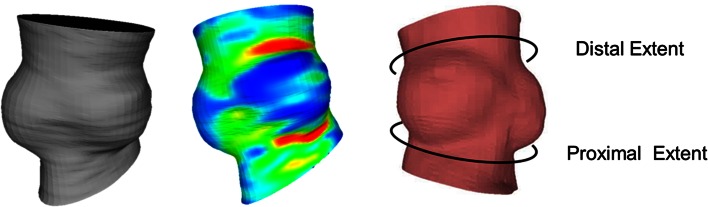
The segmented 3D aortic root surface using Normalized Cuts (*Left*). The segmented 3D surface colored by the Gaussian curvature map per face (*Center*). Proximal and distal extents shown on 3D surface (*Right*)

The aortic root in the CTA volumes was automatically segmented by performing the following steps: first, the structure of interest was detected using thresholding and connected component analysis [16]. The centerline through the ascending aorta and aortic root was determined. Subsequently, high intensities due to calcifications were masked. Finally, the aortic root was represented in cylindrical coordinates and filtered using a 3D Gaussian filter allowing the segmentation of the aortic root using 3D normalized cuts resulting a 3D surface as illustrated in Fig. 3.

### Proximal and distal extents of the aortic root

To identify the region of interest facilitating the detection of the landmarks, we developed a technique that locates distal and proximal extents of the aortic root (Fig. 3). We exploited the shape of the segmented surface and converted this 3D Cartesian surface into a 2D radial map. Based on the aorta centerline, Multiplanar Reconstructions (MPRs) perpendicular to this centerline were calculated. For every slice, the Fourier transform of the radius of aorta surface was calculated. The elliptical shape of the LVOT is expressed by strong second harmonic contributions; the three sinuses are associated with a strong third harmonic contribution of the Fourier decomposition. We analyzed the ratio of the third harmonic and the second harmonic contributions. This ratio enhances the accuracy of the three sinuses detection, minimizing the effect of the elliptical shape of the LVOT. We applied the Laplacian operator to the resulted ratio, producing a signal with two local maxima that represent the proximal and distal extents of the aortic root. The sinotubular junction (STJ), which is the region between the aortic sinuses and where the normal tubular configuration of the aorta is attained, was defined as the detected distal extent of the aortic root.

### Coronary ostia detection

**Fig. 4:**
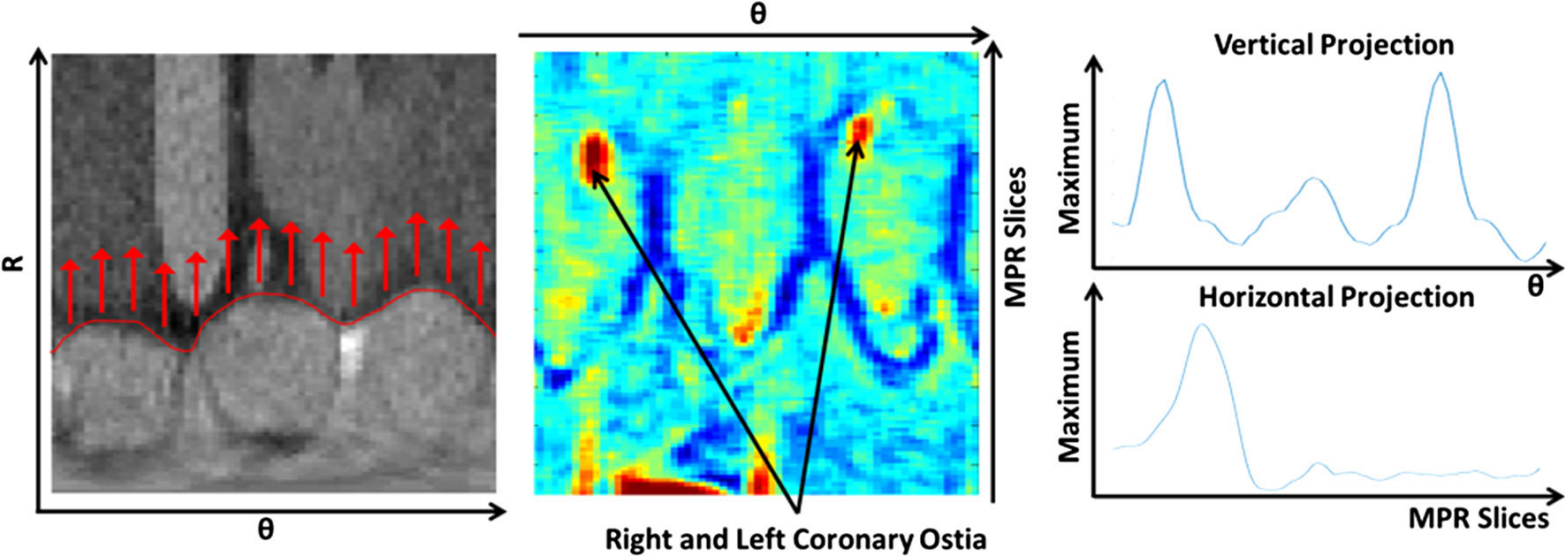
(*Left*) Aortic root image in polar coordinates. The aortic root boundary is shown in *red*. The *arrows* represent the direction for which an average intensity projection images is created (*Center*). The projection image is displayed in the *middle* showing the two local maxima representing the coronary ostia. Two 1-D maximum projection curves were calculated (*Right*) to determine the proximal–distal and angular locations of the coronary ostia

To locate the coronary ostia on the 3D aortic surface, the relative high intensity in the coronary arteries was used as the main feature. To detect the high intensity contributions, an image of the average intensity value of the volume between the segmented aortic surface and a dilated surface was calculated as shown in Fig. 4. Each pixel in this image represents the average intensity along a cylinder starting at the aortic root surface with a length of 2.5 mm. This cylinder has a radius of 0.75 mm. The rows in this image represent the MPR slices and the columns represent the angle around the centerline. The direction of the cylinders is shown as arrows in Fig. 4.

Each pixel in the resulted image is weighted based on its proximity to the distal extent in a Gaussian fashion. The used Gaussian model is centered at the distal extent of the aortic root and spread with a SD of 4 mm in both proximal and distal directions. We used the location of the distal extent because it is close to the sinutubular junction and the coronary ostia, granting the neighboring pixels the possibility to be selected as a coronary ostium and enhance the detection.

Two 1D profiles were created by the projection of the maximum values of the image in both dimensions. The projection in proximal–distal direction generated a profile as a function of the angle (Fig. 4). In this profile, the two distinct local maxima represent the angles of the two coronary ostia. In the proximal–distal direction profile, a single maximum was found, which represents the location of the ostium along the centerline.

### Hinge point detection

The aortic valve annulus represents the narrowest part of the aortic root and is defined as a virtual ring with three anatomical anchor points at the base of each of the attachments of the aortic leaflets. Often, patients have a heavily calcified annulus, disguising these hinge points in CT images. In this section, we present an algorithm that detects the Right Coronary (RC), Left Coronary (LC) and Non Coronary (NC) hinge points. The hinge points are detected using a combination of three 2D maps; a Gaussian curvature map a minimum intensity inward the aortic wall map (MIIAM), and a maximum intensity inward the aortic wall map (MXIAM). These three maps combine the intensity and geometrical based features. The Gaussian curvature of the aorta wall is determined by computing the curvature tensor and the principal curvatures at each vertex of the surface mesh as shown in Fig. 3, this Gaussian curvature is independent from the aortic root centerline and the two sided opened surface in its calculation.

The MIAAM highlights low intensities representing the leaflets. Each pixel in this map represents the minimum intensity along a cylinder starting at the aortic root surface directed inward with a length of 1.5 mm and radius of 0.75 mm. MXIAM is formed in the same manner but only determining the maximum intensity. In the three 2D maps, the y-axis represents the MPR slices and the x-axis represents the angle around the centerline. We derived a single map by multiplying the formed three maps.

Hereafter the combined image is split into three radial tiles representing the three sinuses. Each radial sinus tile represents one sinus and is thresholded at half of the maximum value. Principal components are derived from the thresholded, binary images. The principal component for each radial sinus tile is used as a search direction for the hinge points on the combined map.

**Fig. 5:**
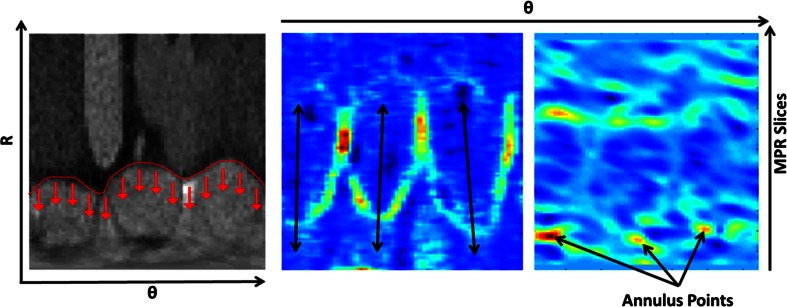
(*Left*) Aortic root image in polar coordinates. The aortic root boundary is shown in *red*. The *arrows* represent the direction of projections. (*Center*) a combined image of minimal, maximal, and curvature images shows the leaflet structure. (*Right*) the Gaussian curvature map shows the convex curvature of the surface

Figure 5 shows an example of a combined map and the three extracted main Eigen vectors. By resampling the combined map data along the sinus Eigen vector, a 1D profile is generated to locate the local maximum, which represents the hinge point. By applying this on each sinus tile, the RC, LC and NC hinge points are detected. The RC hinge point is identified as the most anterior point, while the LC hinge point is the most posterior and left one. The remaining point is considered as the NC hinge point.

### Manual measurements

To validate the accuracy of the automatic landmarks detection, we compared the automatically detected landmarks with manual assessments in the CTA image datasets. Two expert observers (EW and FvK) manually selected the six landmarks using 3mensio software in a 3D curved MPR volume. To reduce interobserver variation due to differences in centerline definitions, the same centerline was used for both observers. The software allowed scrolling though 2D MPR slices to optimally place the landmarks. The annulus plane was defined as the plane connecting the three hinge points. Based on the hinge points, center of the annulus and the 3d orientation of the plane were determined for further analysis. Furthermore, we calculated the radius of the circle connecting the three hinge points. Similar to the annulus plane, the manual STJ plane parameters were determined using three manually selected STJ points.

Based on the landmarks, the sizing parameters that are required in the pre-procedure planning of TAVI were calculated. These sizing parameters include the location and orientation of the annulus plane and the distances from the annulus plane to the right and left coronary ostia. The sizing parameters were calculated for the automatically extracted landmarks as well as for the manually set landmarks.

### Statistical analysis

In the accuracy assessment of the proposed method, measurements based on the manual landmarks annotations were considered reference values. Accuracy of continuous measures, such as annulus radii, and annulus to ostium distances, was assessed using Bland–Altman analysis and the calculation of the intraclass correlation coefficient. The interobserver variation analysis was performed using the same methods.

The accuracy of landmark location detection is performed by the calculation of distances to reference locations were visualized using box-and-whisker plots. The accuracy of the annulus and STJ planes is assessed by determining the center shift and the planar angle between the automatically detected and reference planes. Accuracy was presented for all patients together as well as for the end systole and end diastole and stenotic and non-stenotic subgroups separately. Analyses were performed using MATLAB and SPSS 19.0 and all variables were reported as a mean, SD, and median.

## Results

### Evaluation of landmark detection

**Fig. 6:**
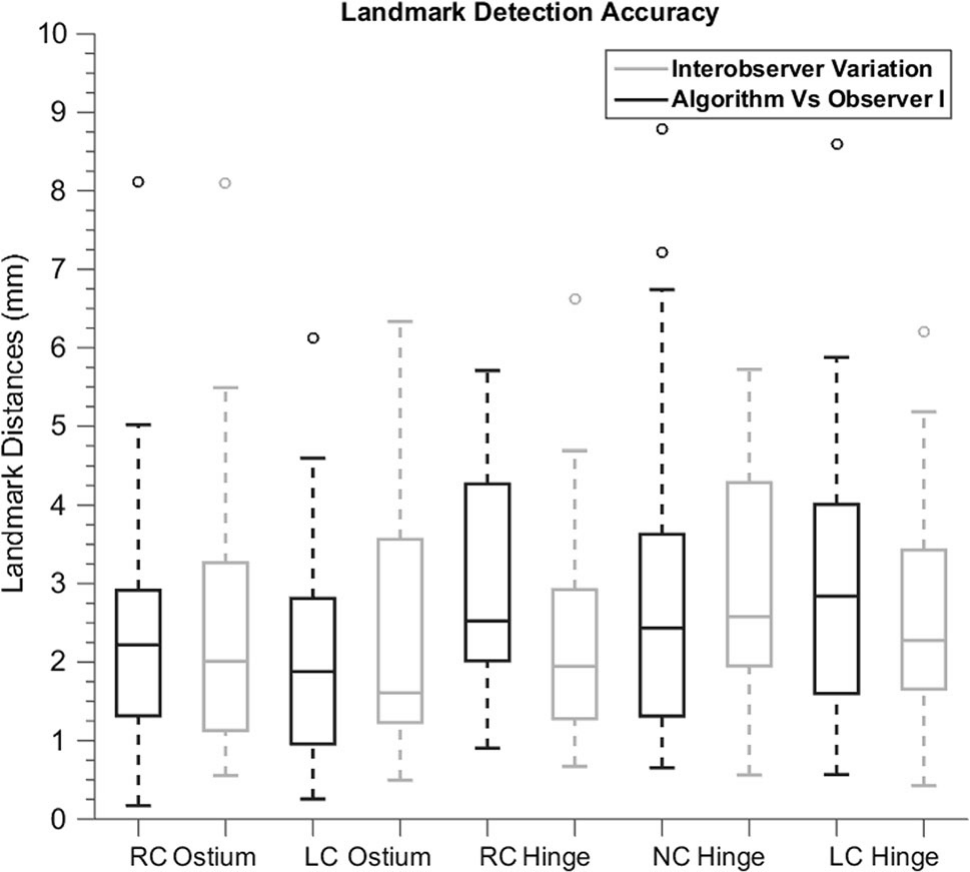
*Box-whisker plot* representing the landmark detection accuracy of the proposed method and the interobserver variation. *RC* right coronary, *LC* left coronary, *NC* non-coronary

The aortic root segmentation was successful in all 40 patients. The accuracy and interobserver variation of the landmark detection is shown in Fig. 6 and Tables 1 and 2. The automated detection of the landmarks had a mean error of 2.66 ± 1.63 mm and 2.96 ± 2.52 when compared with Observer I and Observer II respectively. The mean paired distance of the observers was 2.38 ± 1.56 mm. The STJ has been detected successfully in all images. The distances of the STJ plane center of the automated detected STJ with manually measurements was 2.97 ± 2.87 mm. The average observer paired distance of the STJ center was 2.54 ± 4.02 mm which showed comparable results with the automated STJ center detection. Table 1 also shows the accuracy of the landmark detection and the interobserver variation for all data and for stenotic versus non-stenotic and end diastole versus end systole separated.

**Table 1:** Average, median, and SD of the Euclidean distance between landmark coordinates for the algorithm accuracy and interobserver variation

Measurement error (mm)	Algorithm versus observer I		Algorithm versus observer II		Interobserver variation	
	Mean ± SD	Median	Mean ± SD	Median	Mean ± SD	Median
Right coronary ostium	2.37 ± 1.44	2.22	2.02 ± 1.34	1.65	2.38 ± 1.56	2.01
Left coronary ostium	1.99 ± 1.30	1.88	3.25 ± 4.57	1.95	3.21 ± 4.89	1.61
Right coronary hinge point	3.03 ± 1.48	2.52	3.45 ± 1.89	2.95	2.24 ± 1.26	1.95
Non coronary hinge point	2.84 ± 1.93	2.44	2.86 ± 1.57	2.56	2.96 ± 1.53	2.58
Left coronary hinge point	3.06 ± 1.72	2.84	3.21 ± 1.50	3.36	2.53 ± 1.22	2.28
Overall error	2.66 ± 1.63	2.35	2.96 ± 2.52	2.46	2.67 ± 2.52	2.23
Stenotic patients	2.66 ± 1.60	2.31	3.02 ± 2.68	2.62	2.69 ± 2.73	2.25
Non-stenotic patients	2.66 ± 1.73	2.35	2.76 ± 1.97	2.26	2.60 ± 1.77	2.23
End diastole image volumes	2.57 ± 1.58	2.23	2.75 ± 1.82	2.42	2.48 ± 1.62	2.14
End systole image volumes	2.94 ± 1.76	2.60	3.65 ± 4.05	2.62	3.32 ± 4.35	2.51

The overall Error and different subsets (e.g. stenotic, non-stenotic, end diastolic analysis, and end systole analysis) of the dataset are shown

**Table 2:** The average, median and SD of the annulus angle difference, annulus to ostium distances, annulus center distance, sinotubular junction center distance, angle difference, and corresponding annulus radius for the accuracy of the proposed algorithm and interobserver variation

Measurement error	Algorithm versus observer I		Algorithm versus observer II		Interobserver variation	
	Mean ± SD	Median	Mean ± SD	Median	Mean ± SD	Median
Annulus to ostia distance (mm)	−0.13 ± 2.46	0.10	−0.27 ± 2.63	−0.08	−0.14 ± 2.06	−0.16
Annulus radius (mm)	0.24 ± 0.70	0.16	0.37 ± 0.82	0.46	0.61 ± 0.71	0.64
Annulus center (mm)	1.93 ± 0.90	1.81	2.12 ± 1.02	1.98	1.61 ± 0.90	1.25
Annulus plane (°)	6.86 ± 5.39	6.02	6.34 ± 4.00	5.14	4.69 ± 3.82	3.91
Sinotubular junction center (mm)	2.97 ± 2.87	1.86	3.06 ± 4.15	1.45	2.54 ± 4.02	1.35
Sinotubular junction plane (°)	13.7 ± 14.5	9.1	13.2 ± 22.3	7.5	11.1 ± 15.4	5.0

### TAVI sizing parameters

The average annulus to right and left coronary ostium distances were 17.2 ± 3.5 and 16.7 ± 3.0 mm respectively for the automated analysis and 17.6 ± 3.2 and 16.6 ± 3.6 mm for observer I. The average hinge points circle radius was 12.2 ± 1.4 mm for the automated analysis and 12.4 ± 1.3 for the reference manual measurements (Table 3).

**Table 3:** The mean, median and SD of the sizing parameters (annulus to left, right ostia distance and corresponding annulus radius) estimated by the developed automated algorithm and calculated by the two observers

Measurement (mm)	Proposed algorithm		Observer I		Observer II	
	Mean ± SD	Median	Mean ± SD	Median	Mean ± SD	Median
Annulus to left ostia distance	16.74 ± 3.01	16.77	16.55 ± 3.58	16.37	16.60 ± 3.23	16.59
Annulus to right ostia distance	17.15 ± 3.51	16.56	17.59 ± 3.16	17.5	17.83 ± 3.33	17.29
Corresponding annulus radius	12.20 ± 1.35	11.99	12.44 ± 1.26	12.3	11.83 ± 1.20	11.72

**Fig. 7:**
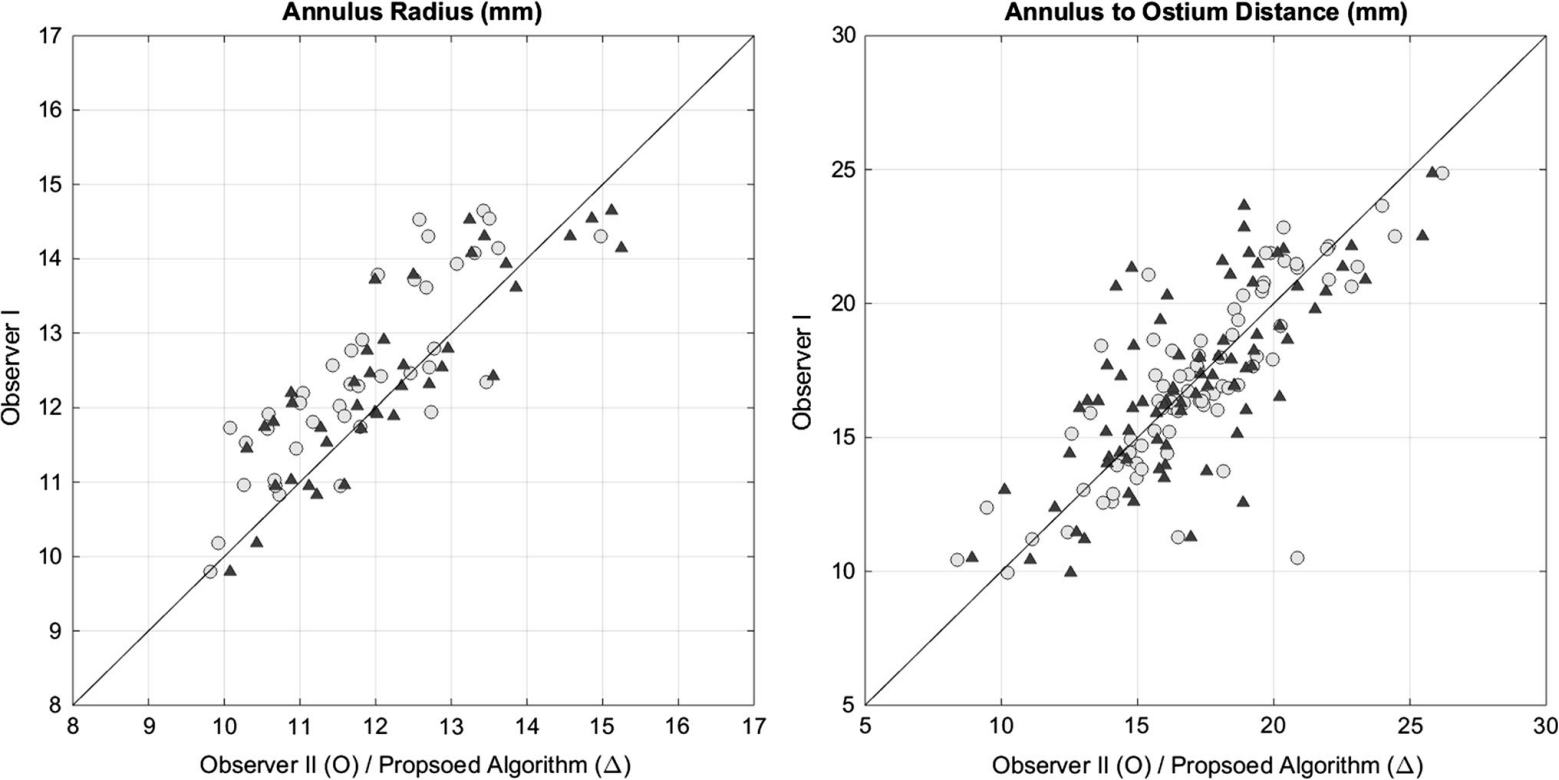
Scatter plots of (*left*) annulus radius of the proposed algorithm/observer II versus observer I (*Right*) annulus to ostium distance of the proposed algorithm/observer II versus observer I

**Fig. 8:**
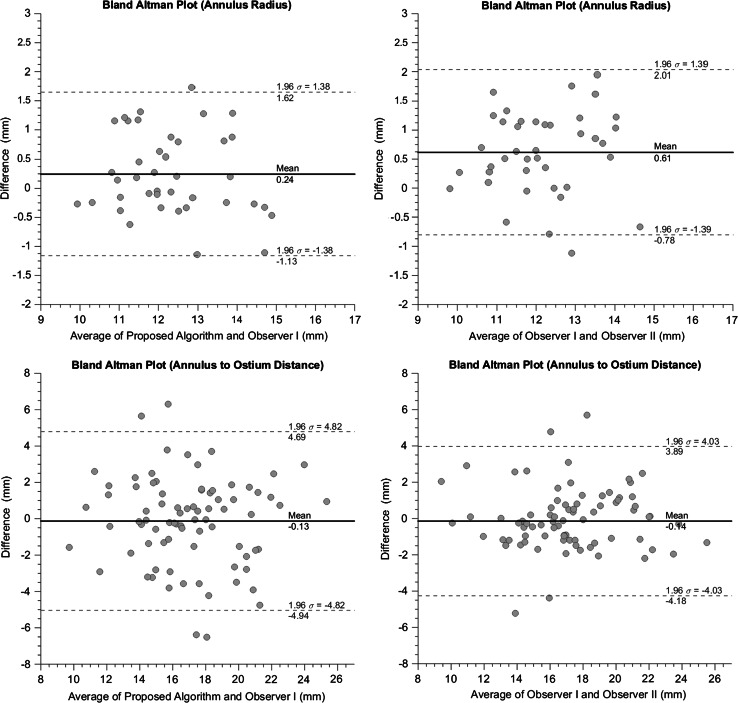
Bland–Altman plot of the proposed algorithm versus observer I (*Left*) and agreement between both observers (*Right*) Annulus radius (*Top*) Annulus to ostium distance (*Bottom*)

The agreement of the automated and manual measures and the interobserver agreement are illustrated in a scatter plot in Fig. 7. The Bland–Altman analyses resulted in a mean paired difference of 0.25 mm for the annulus radius between the proposed algorithm and observer I (Fig. 8). The mean paired difference for the observers was 0.62 mm. For the annulus to ostium distance, the mean paired difference between algorithm and observer I was 0.13 mm where interobserver mean paired difference was 0.14 mm with a narrower limits of agreement.

Intraclass correlation coefficient (ICC) showed strong agreement between observers for both annulus radius and annulus to ostium distance with 0.73 and 0.81. The automated algorithm had a comparable agreement of 0.84 with observer I for annulus radius and had lower agreement of 0.73 for the annulus to ostium distance (Table 4).

**Table 4:** The intraclass correlation coefficient for annulus to ostium distance, annulus radius, and distance between hinge points

Intraclass correlation coefficient	Algorithm versus observer I	Algorithm versus observer II	Interobserver variation
Annulus to ostium distance	0.73	0.68	0.81
Annulus radius	0.84	0.77	0.73

In Table 2, the differences in annulus to ostium distance, annulus radius, annulus center, annulus plane angle, STJ center, and STJ plane angle are shown. Comparison of the automated method with the reference measurements showed a mean paired annulus angle of 6.9° and annulus center mean shift of 1.9 mm. The mean differences between the observers of the annulus angle and annulus centers were 4.7° and 1.6 mm, respectively. The mean paired difference of the annulus to ostium distance was −0.13 ± 2.46 mm, which is comparable to the interobserver mean paired difference of −0.14 ± 2.06 mm.

## Discussion

We presented a fully automated method for detecting landmarks in the aortic root to facilitate automated sizing in preprocedural evaluation of TAVI patients. This method detected the STJ, the two coronary ostia, and three valvular hinge points, which allowed the calculation of clinically important sizing parameters such as the annulus to ostia distance, annulus radius, and annulus angle. Our proposed algorithm has a high accuracy in comparison with manual measurements.

Previous studies presented alternative methods for the detection of the aortic root landmarks on various imaging modalities for TAVI purposes. Zheng et al. [23] introduced a fully automatic landmarks detection in C-arm images using a hierarchical approach by first detecting a global object using marginal space learning with subsequent refinement in a small region under the guidance of specific landmark detection. In the study by Waechter et al. [24], a model based segmentation for CT data was used to locate the coronary ostia and annulus plane. This coronary ostium detection used intensity pattern matching as an extra step for refinement of the ostium location. In their study, the accuracy was not compared with manual interobserver variation, whilst such comparison is an essential constraint for introduction in clinical practice.

Compared to both previous studies, our current method demonstrated similar accuracies in terms of automatically located location to reference location error. Zheng et al. reported worse accuracy for the annulus to ostium distance measurements as compared to the reported accuracies in the present study. None of the previous studies have assessed the interobserver variability, while Waechter et al. [24] could not detect all the coronary ostia.

In general, there was a high agreement between all measurements but there was only one outlier in the agreement between observers for the annulus to ostium distance with a difference of 9 mm. Post-hoc analysis indicated that this may have been caused by the difficulty in depicting the heavily calcified right coronary ostium.

The automatic detection of the hinge points was not straight-forward due to presence of extensive calcifications in the region of the annulus plane and the left ventricle outflow tract in some of the patients. These difficulties are reflected in occasional larger differences between the manual measurements and the automated algorithm (up to 3.1 mm for the hinge points in comparison with coronary points with 2.4 mm).

The detection of the left coronary ostium was more accurate than the right coronary ostium. This may be due to a relatively large left coronary artery diameter compared to the right coronary artery [25]. Moreover, movement of the left coronary artery is limited at 70 % of the cardiac cycle. Moreover, a smaller diameter of the right coronary artery is associated with larger partial volume effects, which lead to a less accurate detection. It is notable that the annulus angle error is not strongly affecting the annulus to ostium distance as shown in Table 2.

The proposed algorithm accuracy was comparable for stenotic and non-stenotic patients with slight larger differences for stenotic patients. We believe that having little or no calcifications for the non-stenotic aortic valve makes the landmark detection more robust. Interobserver limits of agreement at the end systole time phase volumes were narrower than the end diastole volumes. The same trend was observed for the accuracy of the proposed algorithm.

This study suffers from some limitations. The automatic aortic root surface segmentation produced smoothed surfaces, which may affect the accuracy of landmarks. Some of the landmarks are located on strongly bending structural surface locations, which are not manifested in the final detected surface using normalized cut. This could partly explain the differences with manual assessment in the landmarks detection. Our proposed algorithm was evaluated on data acquired with a single CTA scanning protocol with highly controlled contrast administration. It was not evaluated whether the reported accuracy will sustain for large deviations; for example, blood Hounsfield units when using other institutes’ acquisition protocols.

For the assessment of interobserver variation, a single centerline was used. In clinical practice, every analysis is initiated with the generation of a new centerline on which the analysis are based. Therefore, the interobserver variation may be underestimated by using one centerline for two observers.

The proposed algorithm was only validated on stenotic and small number of non stenotic patients. It could be that the proposed algorithm is less accurate in patient populations with deviations in aortic shape, e.g. a pediatric population, patients with Marfan syndrome, and patients with aortic root dilation. Image data from one single medical center and scanner was used in this study. Although there was a large variety in scanned volumes, image to noise ratio, and anatomy, different scanning protocols may require adjustments of the presented algorithm.

## Conclusion

We have presented an analysis pipeline for automated sizing in preprocedural CTA image data of patients eligible for TAVI procedures based on the detection of aortic root landmarks. The accuracy was similar to the interobserver variation in terms of annulus to ostium distance, annulus angle, shift in annulus center, and corresponding annulus radius. Because of the reported accuracy, this automated method is suitable for introduction in clinical practice.
